# What does neighbourhood climate action look like? A scoping literature review

**DOI:** 10.1007/s44168-022-00009-2

**Published:** 2022-05-12

**Authors:** Neelakshi Joshi, Sandeep Agrawal, Shirley Lie

**Affiliations:** 1 School of Urban and Regional Planning, 3-107 Tory, University of Alberta, Edmonton, T6G 2E3 Canada; 2 Leibniz Institute of Ecological Urban and Regional Development, Weberplatz.1, 01217 Dresden, Germany

**Keywords:** Neighbourhood, Climate action, Planning, Bottom-up, Mitigation, Adaptation, Urban ecology

## Abstract

Cities are recognized as an important scale for framing and implementing plans and policies for action on climate change. Within the structure of cities, it is in urban neighbourhoods that climate action becomes tangible and has the potential to engage communities. Despite its importance, scholarly literature has played limited attention to the scale of the neighbourhood as a site for locating climate action. The objective of our paper is to provide an overview of the role of neighbourhoods in leading bottom-up climate action and its implications for urban planning based on a qualitative scoping review. Our findings indicate that neighbourhoods are conceptualized as a physically bounded scale for climate action as well as a web of social networks and relationships enabling this action. Neighbourhood climate action aims to achieve neighbourhood scale sustainability and resilience by engaging with residents, municipalities, local academic institutions, neighbourhood associations and non-governmental agencies. Scholars engage with a wide range of concepts like place-based attachment and social mobilization as well as established practice-oriented tools in defining and measuring neighbourhood climate action. However, the neighbourhood scale struggles with limited resources and power in creating sustained climate action as well as in engaging with and addressing socio-economically marginalized communities.

## Introduction

More than half of the world’s population lives in cities and directly or indirectly influences a majority of the global greenhouse gas (GHG) emissions (United Nations, 2019). Large population concentrations also make cities vulnerable to the impacts of climate change (IPCC, 2018; Pelling, 2003). Cities are thus recognized as an important scale for framing and implementing plans and policies for action on climate change (Bulkeley et al., 2011). Climate action is a broad term that covers both mitigation and adaptation efforts to address climate change and is covered under Goal 13 of the United Nations Sustainable Development Goals (United Nations, 2015). To respond to the global call to reduce GHG emissions and prepare for climate change, cities across the globe have formulated climate action plans (Guyadeen et al., 2019; Hughes, 2019). However, given the magnitude of action required to address climate change, there is a growing consensus among scholars to engage with and magnify the scale and scope of bottom-up and community-led local climate action (Cloutier et al., 2018; Seyfang & Smith, 2007).

Urban neighbourhoods are an important scale of physical and social organization within the structure of cities (Rohe, 2009). It is in neighbourhoods that climate action gets “contested, deconstructed and reconstructed” (Wittmayer et al., 2014). Further, the neighbourhood scale is easily recognized by urban residents as a place for participation and experimentation around climate action (Rohe, 2009). Neighbourhood climate action moves beyond the individual or the state, and focuses on the community (Aylett, 2013; Joshi et al., 2022). Concepts like low-carbon localism are beginning to define and create a framework for bottom-up climate action in urban neighbourhoods (Bradley et al., 2017). Further, the COVID-19 pandemic had restricted people’s movement and brought back the focus on their immediate neighbourhood surroundings (Joshi et al., 2020; Moreno et al., 2021).

**Fig. 1:**
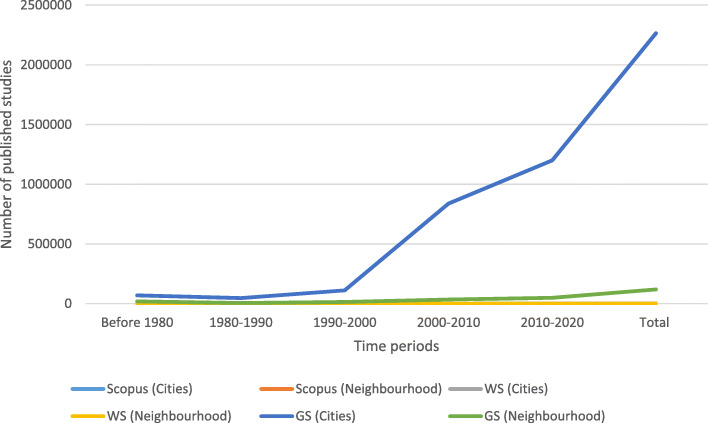
Comparison of the number of published research article on “cities + ‘climate action’” vs ‘neighbourhood + climate action’ between 1980 and present

The role of cities in addressing climate action has increasingly been the subject of scholarly enquiry (Bulkeley et al., 2011; Hölscher & Frantzeskaki, 2021; Wolfram, 2017). Urban studies are building on the lessons learnt from sustainable urbanism and moving towards carbon urbanism (Hughes et al., 2020; Long & Rice, 2019). In comparison, limited attention has been paid to the scale of the urban neighbourhood as a site for addressing GHG emissions (Pulselli et al., 2018; Welegedara et al., 2021) and locating climate action (Bradley et al., 2017; Gilderbloom et al., 2017). To understand the scale of this gap, we mapped research articles between 1980 and 2020 on three research databases, namely Scopus, Web of Science (WS) and Google Scholar (GS), to compare the volume of research on ‘cities + climate action’ against ‘neighbourhood + climate action’. Figure 1 and Table 1 show that post 2000, there has been a substantial increase in research output on cities and climate action. Research on neighbourhoods has also begun receiving attention. However, it is significantly low when compared to cities.

**Table 1:** Comparison of the number of published research article on “cities + ‘climate action’” vs ‘neighbourhoods + climate action’ between 1980 and present

	Before 1980	1980-1990	1990-2000	2000-2010	2010-2020	Total
Scopus (cities)	5	13	20	226	1809	2073
Scopus (neighbourhood)	2	1	2	20	136	161
WOS (cities)	15	6	29	237	2089	2376
WOS (neighbourhood)	31	0	3	12	144	190
GS (cities)	68,700	46,200	111,000	838,000	1200,000	2263,900
GS (neighbourhood)	18,700	5000	13,700	33,700	48,500	119,600

We argue that there is a need to advance the knowledge on neighbourhood climate action on three accounts. First, climate action has added a focus and momentum to the broad agenda of sustainable development by focusing on scaling up mitigation and adaptation efforts within a limited time frame (Soergel et al., 2021; Tosun, 2022). A multi-scalar perspective helps understand actions that can be taken at international, national, regional and local scales to act on climate change (Bulkeley, 2021; Bulkeley & Newell, 2010). Urban neighbourhoods are one such site of local, bottom-up climate action. While there is well-synthesised knowledge on neighbourhoods as sites of locating sustainable urban development (Grazieschi et al., 2020; Luederitz et al., 2013), locating climate action at the neighbourhood scale is an attempt to further push the boundaries of the multi-scale perspective towards addressing climate change. Second, the focus and momentum created by the urgent call for climate action has opened up room for experimentation and financing for supporting bottom-up climate action (Broto & Bulkeley, 2013; Evans & Karvonen, 2014; Heiskanen et al., 2015). However, we do not know enough about the knowledge, skills, capacities and willingness at the neighbourhood scale to participate in bottom-up climate action. Finally, as societies attempt to transition and transform towards a low carbon future, new modes of governance and power sharing might be needed to create and sustain bottom-up climate action (Anguelovski, 2015; Brand, 2016; Bruch, 2017; Kivimaa et al., 2017). Exploring neighbourhoods as sites for climate action is one possible venue to explore these ideas (Joshi et al., 2022).

Against this backdrop, we present a scoping review of the literature on neighbourhood climate action. We aim to synthesise existing research with the aim of creating broad contours for understanding the concept of neighbourhood climate action by answering the research question: what constitutes neighbourhood climate action? Based on 68 research articles addressing the topic, we elaborate upon the scale of the neighbourhood as a place for situating bottom-up climate action within the city. The “Review methodology” section elaborates upon the review methodology adopted for selecting and analysing research articles. The “Key findings” section discusses the key findings in terms of conceptualizing neighbourhoods, types of mitigation and adaptation actions and the governance structures and actors involved. Finally, the “Conclusion and future directions of research” section highlights the key gaps that emerged from the literature review as well as identifies future areas of research at the neighbourhood scale. The synthesised findings are useful for researchers as well as social groups in identifying the opportunities and challenges associated with the neighbourhood scale as a site for situating climate action.

## Review methodology

Literature reviews are a tool for knowledge advancement in academic literature. They capture both the breadth and depth of existing knowledge and bring out the gaps to lay the foundation of future research (Xiao & Watson, 2019). Among the multiple methodologies that exist to conduct a literature review, the choice is determined and defined by the nature of the research question and the method adopted to collect, evaluate and present information (Paré et al., 2015). Given the broad nature of our research question and limited comprehensive knowledge on the topic, we adopted a scoping review approach to provide a snapshot of research on the subject (Arksey & O’Malley, 2005; Colquhoun et al., 2014).

We adopted the five step approach for conducting a scoping review laid forth by (Arksey & O’Malley, 2005). This involves identifying the research question, identifying relevant studies, establishing a study selection criterion, charting the data and collating, summarizing and reporting the results.

The broad research question that we intend to address is: what constitutes neighbourhood climate action? We began our search with the Boolean string “neighbo*rhood*” AND “climate action” on three research databases, namely, Scopus, Web of Science (WS) and Google Scholar (GS). GS was intentionally added for making the search comprehensive and to include grey literature sources. This yielded 20 results on Scopus, 26 results on web of science and 10730 results on GS as of October 2020. As GS results were substantially large in number, we constrained our search to the first 100 results, sorted in the order of relevance.

We exported the first set of 146 search results to a web-based literature review manager called Covidence. We found Covidence to be a convenient platform for screening our search results as it allowed multiple researchers to review and vote on inclusion or exclusion of studies. It also automatically identified removed 28 duplicate research items. The inclusion criteria set for the research were English language articles from countries of the Global North between the years 2000 and 2020. Further, the conceptualization of neighbourhoods as a unit of human settlements in cities was used. Countries of the Global North were purposively selected for this scoping review for a similar conceptualisation of the concept of neighbourhood, neighbourhood planning and climate action. Studies published after the year 2000 were selected as Scopus and WS did not show any studies before that period.

For the next stage, we conducted a title and abstract review based on the inclusion criteria. Each research article was reviewed and voted on by two researchers. Conflicts in voting, if any, were discussed based on the inclusion criteria and finally a total of 61 research articles were taken up for full text review.

**Fig. 2:**
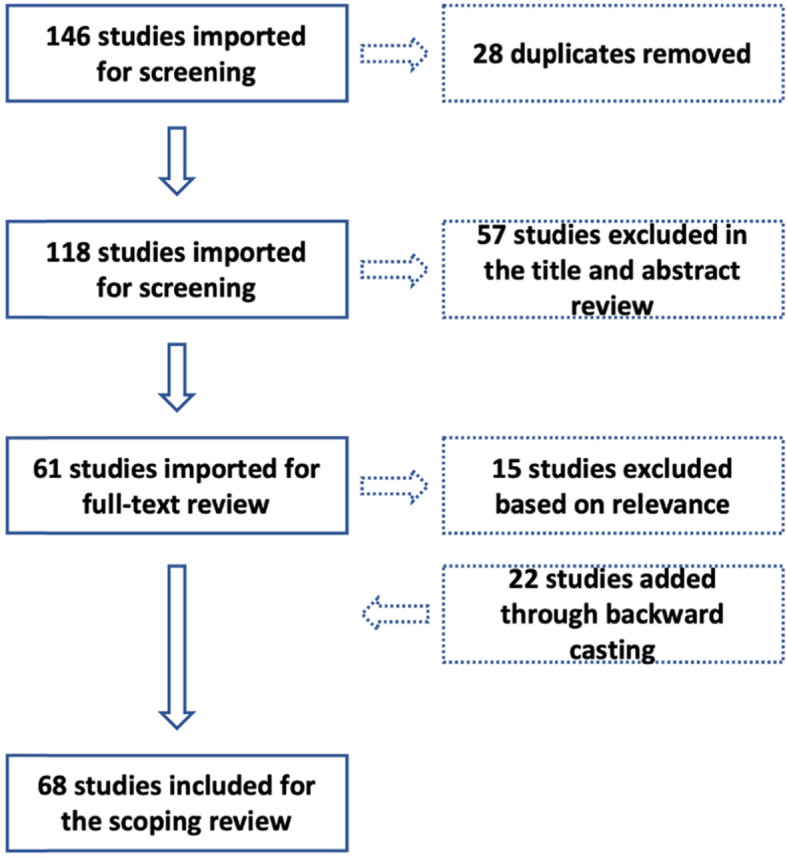
Workflow diagram for including and excluding studies for the scoping review

Each of the 61 review articles was read by at least two researchers. Through this process, we further eliminated 15 research articles but added 22 articles through the process of backward casting by identifying articles from the existing reference lists. A total of 68 research articles were thus taken up for full text review. Among them were 59 peer-reviews papers, 4 conference proceedings, 3 book chapters, 1 working paper and 1 doctoral dissertation. Figure 2 summarises the workflow adopted for identifying and selecting research articles for this study.

We extracted both quantitative and qualitative data from the research articles. Quantitative data, extracted using Microsoft Excel, included a word count of keywords, countries, and regions where the study was conducted, nature of climate action (adaptation or mitigation or both), nature of the neighbourhood (existing or new) and nature of study design (qualitative, quantitative, or mixed). Qualitative data, extracted using NViVo, included inductively coding the articles for recurring themes. These included the conceptualisation of neighbourhood and its relevance as a scale for climate action and conceptualisation of climate action. We also used the qualitative analysis to elaborate upon the theoretical and conceptual foundations of neighbourhood climate action, research methods adopted, actors involved and finally the challenges highlighted by the existing research. The following section presents the results of our analysis.

## Key findings

**Fig. 3:**
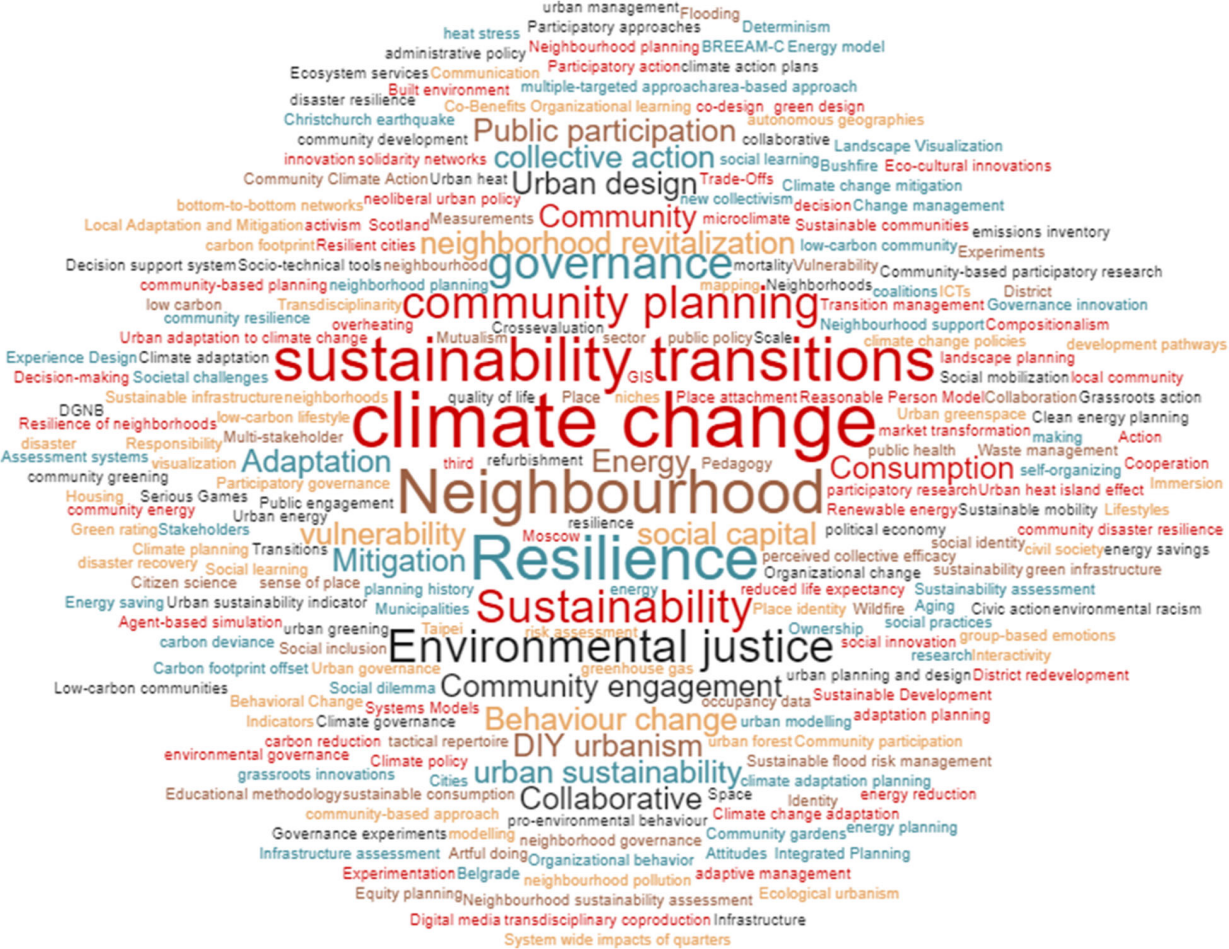
Results of the keyword analysis

A keyword analysis of 68 research articles resulted in 296 keywords. Climate change (16) and neighbourhood (8) were the two most prominent keywords. This was followed by sustainability (4) and sustainability transitions (4) and resilience (5), vulnerability (3) and adaptation (2), indicating that these are foundational concepts upon which neighbourhood climate action rests. Governance (10) and planning (16) appear in multiple iterations, e.g. climate change governance, collaborative governance, neighbourhood planning, community planning, etc. Fig. 3 graphically presents the keyword analysis, and the “Conceptualizing neighbourhoods and their role in climate action” to “Challenges” sections elaborate of key themes of neighbourhood climate action, identified through inductive coding.

### Conceptualizing neighbourhoods and their role in climate action

Neighbourhood is defined as the fundamental building block of the city (Rohe, 2009). Neighbourhoods function like cells within the organism of a city (Castrignanò & Landi, 2013). Two distinct aspects emerge in the conceptualization of the neighbourhood from this literature review. First, the neighbourhood as a physical entity composed of a physical boundary, buildings, streets, trees and other physical infrastructure. Second is the neighbourhood as a social entity that provides space for social interactions, relationships and collective identities to form. The physical and social aspects come together to create an understanding of the neighbourhood as a community of place defined on a Euclidean map (Taylor Aiken, 2014). However, within a neighbourhood, heterogeneous values and communities of interest may exist (Taylor Aiken, 2014). Neighbourhoods are thus an intricate interaction between buildings, transport, green spaces, human activity and structures (Evola et al., 2016).

The scale of the neighbourhood is small enough to demand its own urban design and public policy, yet big enough to create an impact at the city scale (Oliver & Pearl, 2018). It is at the scale of the neighbourhood that large scale global problems are “contested, deconstructed and reconstructed” (Wittmayer et al., 2014). The neighbourhood is an important site of public participation and civic action as the scale is easily identifiable to its residents and provides a space around which everyday life is organized (Rohe, 2009; Rowlands, 2011). Local action has the potential to reshape citizenship, change power relations, increase participation in decision making and encourage democratization and transformation in cities (Anguelovski, 2015). Historically, the neighbourhood scale has received limited attention in terms of power and resources (Rohe, 2009) in comparison to the city. However, action on climate change provides an opportunity to explore the neighbourhood as a site for bottom-up action. A focus on neighbourhoods shifts the attention of climate action from the individual or the state, to the community (Aylett, 2013). It is an opportunity to re-imagine local capacities in the face of capitalist globalism and question if the local is a mere product of global forces or has its own culture and identity (Massey, 2004).

The challenges brought about by climate change present an opportunity to explore new models, structures and organizations at the neighbourhood scale (Rowlands, 2011). On the one hand, the physical design of the neighbourhood can influence and support low carbon habits like walking or taking public transport (Rohe, 2009). Decisions taken at a neighbourhood scale, like the amount of green space, influence the community’s response to climate shocks and stresses (Uda & Kennedy, 2018).

On the other hand, social capital existing in a neighbourhood presents an opportunity to build partnerships and networks and open up new ways of problem solving around climate action (Slater & Robinson, 2020). Neighbourhood scale actors promoting climate action are intimately connected with city, regional, national and international actors in negotiating their geographical agency and responsibility (Shaw et al., 2018). However, group identity and a sense of belonging to a neighbourhood are important to encourage participation in neighbourhood-based collective action around climate change (Rees & Bamberg, 2014). In such neighbourhoods, people often feel a greater magnitude of responsibility towards their neighbours and the neighbourhood (Massey, 2004; McGee, 2011) and provide immediate help in case of disasters or emergencies (Aldrich & Meyer, 2015).

### Climate change mitigation and adaptation at the neighbourhood scale

Among the 68 papers that we reviewed, 16 dealt with neighbourhood mitigation actions, 18 dealt with adaptation, 18 addressed both and 16 papers presented underlying theoretical concepts.

Neighbourhood scale action in addressing climate change mitigation typically dealt with issues around energy efficiency of buildings (Fisher & Irvine, 2010; Westerhoff et al., 2018), low-carbon mobility solutions (Shelton, 2008), renewable energy production and planning (Aylett, 2013; Evola et al., 2016; Hettinga et al., 2018), waste management (Pulselli et al., 2018) and water efficiency (Scarfo, 2011). Some initiatives intend to motivate sustainable consumption cultures (Middlemiss, 2008) and to develop skills needed for leading low carbon lives which include activities like bike repair workshops and renewable energy workshops (Büchs et al., 2012). GHGs were often the unit for measuring the impact of actions and projects advocated for low-carbon impacts (Pulselli et al., 2018). Literature on mitigation efforts often overlaps or builds upon existing literature on neighbourhood scale sustainability (Wittmayer et al., 2014). Neighbourhood scale climate action creates a space for social mobilization for climate action by aiming to build public support for climate action, creating capacity as well as engaging citizens to co-create and co-implement mitigation projects (Westerhoff et al., 2018).

Literature focusing on neighbourhood scale adaptation typically dealt with heat stress (Guardaro et al., 2020; Maragno et al., 2020) and flooding (Meyer et al., 2018) as critical focus areas. As adaptation is context specific, certain studies also dealt with place specific issues, e.g. wild fire adaptation (McGee, 2011). Neighbourhood scale adaptation projects aim to build collective adaptive capacity, both physical and social, among residents to prepare them for unpredictability and respond to disturbances (Cretney & Bond, 2014). Projects like neighbourhood greening and community gardens presented an opportunity for addressing both mitigation and adaptation goals (Cloutier et al., 2018; Klerks et al., 2019; Shelton, 2008). Urban greening aims to improve quality of life by incorporating natural elements into built environments (Cloutier et al., 2018). However, challenges remain in mainstreaming small scale urban greening projects (Cloutier et al., 2018). Adaptation studies often built upon the concept of neighbourhood resilience. Neighbourhood resilience is the ability of a neighbourhood to deal with physical, social and political stresses and disturbances resulting from climate change (Andrew et al., 2020; Castrignanò & Landi, 2013; Guardaro et al., 2020). Adaptation thinking has gradually developed from highlighting global and national macroeconomic markets to the locally assessed susceptibility and resilience within socio-ecological systems (Groulx, 2017). Neighbourhood resilience is a foundation upon which climate change adaptation goals can be built (Kwok et al., 2019). To bridge the action on mitigation and adaptation, certain scholars engage with the socio-ecological systems thinking and explore avenues like neighbourhood greening (Dieleman, 2013; Groulx, 2017).

Neighbourhoods, particularly the new neighbourhoods, present an opportunity to be designed and modelled for a low carbon footprint (Wang et al., 2016). Leadership in Energy and Environmental Design for New Neighbourhoods (LEED ND) and Building Research Establishment Environmental Assessment Method for Communities (BREEAM C) are examples of two performance-based tools that work on an indicator-based approach in designing sustainable neighbourhoods (Reith & Orova, 2015; Wang et al., 2016). Within existing neighbourhoods, GHG footprint mapping tools help residents to measure and manage their consumption patterns (Jones et al., 2018). Some positive impacts of mitigation action can further address adaptation efforts to combat energy shortage, heatwaves and extreme precipitation (Uda & Kennedy, 2018). However, some scholars argue that the focus on the neighbourhood as a sustainability product misses the discussion on the process of sustainability and deeper community engagement (Oliver & Pearl, 2018). Further, other scholars point out that the scale of the city as well as individual buildings have gained attention in terms of sustainability and carbon accounting tools, whereas the scale of the neighbourhood has largely remained unexplored (Palermo et al., 2018; Pulselli et al., 2018). To measure neighbourhood resilience, Kwok et al. (2019) suggest combining both scientific and local knowledge gained from neighbourhood-level stakeholders and city-level authorities.

### Key theories and concepts

In this section, we elaborate upon the underlying theories and concepts that researchers engage with when exploring climate action at a neighbourhood scale. Neighbourhood climate action shares a similar concepts foundation as bottom-up climate action. Our review illustrates how these foundational concepts are applied at a neighbourhood scale and provide analytical sharpness to the concepts of bottom-up climate action.

#### Place attachment

Place attachment is an important aspect in inspiring neighbourhood climate action (Devine-Wright, 2013). Place attachment is an emotional connection between people and place that can influence human behaviours and responses toward climate change (Devine-Wright, 2013; Dulic et al., 2011; Groulx, 2017). This concept can act as both an enabler and a barrier in undertaking adaptation and mitigation efforts. On the one hand, place attachment helps boost residents' involvement in neighbourhood projects (Kwok et al., 2019). Community groups share the same value over the same places perceive adaptation as protection to their landscapes that triggers trust and collective action (Groulx, 2017). Place attachment encourages residents to spend more time to connect with others and together watch their neighbourhoods evolve (Anguelovski, 2015). Participating in community-based adaptation planning can further improve residents' familiarity with climate change impacts on specific places. On the other hand, place attachment sometimes leads to residents’ misperception of government policies and a resistance to change (Groulx, 2017). A failure to recognise emotional bonds that people have with a particular place might lead to community resistance (Devine-Wright, 2013). Thus, understanding place-based assets and susceptibilities is critical in developing adaptation strategy at the local scale by both residents and the government (Groulx, 2017).

#### Mutualism

Place-based attachment often lends itself to mutualism. Mutualism is defined by Rowlands (2011) as “collective action, pooling resources and obtaining an outcome which is greater than the sum of the parts”. Cooperative housing models as means of providing affordable housing at a neighbourhood scale are cited as a successful example of mutualism in practice. Mutualism is proposed as a concept to re-imagine neighbourhoods, especially as they gear to take action on climate change. Finally, social neighbourhood identity, social bonding, trust, perceived efficacy of collective action and group-based guilty consciousness related to climate change plays a role in determining participation in neighbourhood climate action (Hielscher et al., 2011; Rees & Bamberg, 2014). Social bonding and community belonging can boost residents’ involvement in collective actions (Anguelovski, 2015). “Bottom-to-bottom networks” are manifested within the neighbourhood where people connect and undertake such actions for environmental renewal projects. These networks help neighbourhood actors address local challenges using existing resources, creativity and intuition (Anguelovski, 2015).

#### Social capital

Social capital is another dominant concept when discussing neighbourhood climate action. Social capital at a neighbourhood scale refers to the benefits individuals derive from being part of a neighbourhood network (Purdue, 2001). Neighbourhood organizations are often found to be leaders in building and maintaining social capital in a neighbourhood (Ruef & Kwon, 2016). This form of social capital is seen as an ideal approach to enhance adaptive capacities of communities toward disasters since the impacts resulting from disasters may affect the resources rooted in social networks (Kwok et al., 2019). Social capital is an underlying need for effective social mobilization in the neighbourhood around climate change (Westerhoff et al., 2018).

#### Social learning

Social learning as a product of collaborative problem solving is a desired outcome of climate action at a neighbourhood scale (Evers et al., 2016; Slater & Robinson, 2020; Stevenson & Petrescu, 2016). The dialog and conflict generated among groups in addressing climate action help people understand each other better (Evers et al., 2016) as well as create culture and values needed to achieve climate action (Slater & Robinson, 2020). Stevenson & Petrescu (2016) indicate that social learning is important to raise people’s awareness, develop community capacities and increase neighbourhood resilience. Additionally, they emphasizes that social learning with the intention to strengthen social capital at neighbourhood level could be achieved through collaborations between residents and academics, which often come in the form of research.

#### Other concepts

Some scholars press on the need to recognise the unique position of each neighbourhood based on its history (Elwood, 2002), socio-economic conditions (Passe et al., 2020) and urban legislative framework (Bradley et al., 2017). Neighbourhood environment history (Kellogg, 2002), for example, holds the knowledge of urban nature and ecosystem. By considering environmental history, it is expected that neighbourhood planning could have a strong information background and enhance the sense of place through ecological resources utilization and motivate residents to engage in neighbourhood adaptation projects.

### Methods employed

This section discusses the methods adopted in research on neighbourhood climate action. Fifty-one out of 68 papers adopted a qualitative approach, 12 adopted a quantitative approach and 5 papers adopted a mixed methods approach.

Qualitative research designs were dominant in the research papers addressing neighbourhood climate action. Key-informant interviews, focus groups discussions and document reviews were largely employed for data collection. Multiple researchers adopted action research designs involving neighbourhood residents, researchers, city representatives, urban planners, local NGOs and local businesses (Hendricks et al., 2018; Hettinga et al., 2018; Hirsch et al., 2011; Wittmayer et al., 2014). Certain research projects stretched the actor constellations to engage particular demographic groups like senior citizens and school students (Meyer et al., 2018). Action research designs were found to be particularly helpful generating new, bottom-up data at the neighbourhood scale (Hendricks et al., 2018; Hettinga et al., 2018). Community-based experimentation, where residents could participate and reflect on small scale climate action projects, is another emergent research design (Cloutier et al., 2018; Dieleman, 2013; Elwood, 2002; Guardaro et al., 2020; Simíc et al., 2017). Experimentation was also useful in understanding the role and capacity of civic actors and community organizations (Elwood, 2002; Kivimaa et al., 2017).

Among quantitative papers, the focus lies on quantitative assessment of GHG emissions at a neighbourhood scale (Jones et al., 2018; Pulselli et al., 2018), developing an indicator-based system to design low carbon neighbourhoods (Wang et al., 2016) and designing decision support for neighbourhood and city organizations (Hettinga et al., 2018). Scenario development tools are useful for existing neighbourhoods to understand aspects that need retrofitting to reduce GHG emissions (Evola et al., 2016). Further, researchers stress on the need to make data easily accessible and understandable, for example, through innovative visualisations (Pulselli et al., 2018). Quantitative methods were also used to evaluate the social determinants of neighbourhood climate action. For instance, Rees and Bamberg (2014) employ a quantitative survey among 538 city residents to understand how the motivation to participate in neighbourhood-based climate protection is determined by social identity, perceived collective efficacy and group-based emotions.

A limited number of papers adopted a mixed research design. Given the physical and social dimensions of neighbourhood climate action, Passe et al. (2020) highlights the merit of a mixed methods approach. Mixing expert quantitative interviews with qualitative neighbourhood surveys is one example (Kennedy et al., 2013). Dulic et al. (2011) compare quantitative results from a survey taken by students before and after trying a game prototype on climate change impacts and compare it against qualitative interviews with subject experts. Further, integration of spatial analysis tools along with quantitative or qualitative methods is useful in generating context-specific granular data (Maragno et al., 2020; Shelton, 2008).

### Governance structures and actors involved

Given the complex nature of climate action, new constellations of actors and new structures of power are emerging at the neighbourhood scale (Aylett, 2013). Recognising the role of neighbourhoods in addressing climate change requires their integration in multi-level governance and scalar politics (Dieleman, 2013; Shaw et al., 2018). This is important as vagueness on how the actors and their role may delay action on climate change (Guardaro et al., 2020). Among the papers that we reviewed, a number of actors such as residents, neighbourhood organizations, city representatives, urban planners and architects, NGOs and researchers were largely engaged with projects on neighbourhood climate action. Some researchers highlighted the need to involve vulnerable populations within a neighbourhood (Passe et al., 2020) as well as local businesses (Murota, 2014) and institutions like schools or youth organizations (Meyer et al., 2018; Simíc et al., 2017).

Projects based on neighbourhood climate action can broadly be divided into three categories:

- City government-led project: These are the projects where residents are invited to participate in various capacities. The nature of participation varies from mere consultation to co-production (Rohe, 2009). Government led programs often have better financial and knowledge resources but need to partner with the residents for deeper and wider project impacts (McGee, 2011; Meyer et al., 2018). Engaging residents in adaptation strategies helps build physical and social community resilience to hazards as well as develops a relationship between governments and residents (McGee, 2011). Civic actors tend to act as a bridge between residents and the municipality by making them work collaboratively rather than confrontationally (Cloutier et al., 2018; Elwood, 2002; Kivimaa et al., 2017). Further, moving from a centralised to a diverse and decentralized governance structure builds resilience against change and ensures continuity (Dieleman, 2013).
- Projects initiated by neighbourhood residents or citizen organizations: Neighbourhood organizations and homeowner associations are often successful in forming durable partnerships with residents in comparison to external agencies (Andrew et al., 2020; Aylett, 2013; Elwood, 2002). Further, they help in building ‘communal social capital’ (Purdue, 2001) within their communities by bringing people with similar and dissimilar interests together. Community-based organizations also have the potential to utilize ‘collaborative social capital’ (Aylett, 2013; Purdue, 2001) based on their relationship with external institutions and organizations. However, local change agents express frustration over a lot of expectation around climate action but very limited resources or agency to do so (Smith et al., 2013). Rowlands (2011) suggests replacing the existing top down structure of neighbourhood management with small scale neighbourhood trusts involving residents.
- Researcher or NGO led projects: These are often designed around action research or experimentation. Though often short term in nature, these projects are a chance to build or strengthen valuable social infrastructure within neighbourhoods that can address climate change beyond the span of a pilot project (Klerks et al., 2019). Meyer et al. (2018) present an interesting constellation of actors for a participatory action research project that included high school and university students, community activists and researchers. The project provided a co-learning opportunity where students and researchers learnt nuances of community action research and community activists learnt tools and methods for assessing and addressing climate change impacts in the neighbourhood. Co-production, as a means of generating knowledge around climate action, is also gaining ground in research and policy development of the built environment (Stevenson & Petrescu, 2016).

With the push and pull of power and responsibilities at the neighbourhood scale, collaborative governance structures, similar to those adopted in successful neighbourhood revitalisation projects, are coming to the fore (Elwood, 2002; Rohe, 2009). Cross-sector collaborations attempt to engage community members, government officials, business owners and NGOs (Simo & Bies, 2007). Cross-sector collaborations would have a high success rate if they are driven by dedicated stakeholders and champions. Further, devolution of power to the neighbourhoods, as is being done in the UK, presents initial evidence on how residents often favour a preservation of local environment, character and social well-being over a pro-growth agenda (Bradley et al., 2017). Experience from projects designed with neighbourhood residents can help identify and assess the correlation between potential risks and weak infrastructure within their neighbourhood (Hendricks et al., 2018). Partnering with community groups also becomes essential to develop a constructive climate policy, as well as to produce effective engagement strategies that are best suited for policy makers and community residents (Hirsch et al., 2011).

### Challenges

The scale of the neighbourhood presents exciting opportunities to address the challenges of climate change. However, literature recognises multiple challenges associated with scale, time frame, perspective and data for successful and sustained climate action at the neighbourhood scale (Kellogg, 2002). We summarise them under four headings:

- Social challenges: Wittmayer et al. (2014) argue that the scale of neighbourhood as a cohesive geographical and social entity cannot be taken for granted before designing local scale climate action. Individual social interest can influence potential volunteers to be engaged in supporting neighbourhood initiatives or stay inactive (Krebs et al., 2013). Similarly, a social capital approach may overlook certain community groups who do not belong to the dominant neighbourhood social network (Kennedy et al., 2013; Kwok et al., 2019). There is a risk of social networks being exclusionary and exercising their powers over others resulting in loss of trust and participation from the residents (Ruef & Kwon, 2016). Further, new suburban neighbourhoods might have limited or no experience with community networks or environmental activism (Kennedy et al., 2013; Smith et al., 2013). Dieleman (2013) points to a need for developing teaching and training tools to build collaboration among residents.
- Data challenges: Multiple authors point towards the lack of neighbourhood scale data collection and analysis systems needed to design effective policies and programs. Localized and granular data at neighbourhood scale data can help in designing more tailor made plans and policies for mitigation and adaptation (Passe et al., 2020; Wang et al., 2016). There is a need for neighbourhood scale data to develop decision support systems. These include neighbourhood GHG accounting tools that can help quantify and visualize the scale of action needed to mitigate climate change (Pulselli et al., 2018). Researchers notice the interconnectedness of multiple intricate systems in mapping and visualizing climate change impacts in a neighbourhood like tree canopy concentration and storm water drainage (Shelton, 2008). Tools are also needed to assess neighbourhood energy demands as well as demonstrate the quantifiable impact of building retrofits or mixed-use developments (Palermo et al., 2018). However, collecting data at a neighbourhood scale can be challenging, particularly human use and behaviour data, because of time, resources and privacy concerns (Passe et al., 2020).
- Power and resource challenges: Local change agents point towards the mismatch between the expectation with regards to climate action and the limited resources or agency to do so (Smith et al., 2013). While neighbourhood climate action creates high expectations from community groups, they often have limited power and mandate to start and sustain long term projects around climate action (Büchs et al., 2012; Lufkin & Rey, 2014; Taylor Aiken, 2014). One example is the tension that neighbourhood groups face when negotiating space and resources for community gardens in a city against developers and city governments (Shaw et al., 2015). Here, the collective and collaborative models that underline neighbourhood climate action are juxtaposed against capitalistic and individualistic societal realities (Elwood, 2002; Rowlands, 2011).
- Continuity challenges: Collaborative action at a neighbourhood scale is a process that requires time to build (Guardaro et al., 2020). However, often neighbourhood scale projects are short- lived in nature. While experimentations and action research projects are important, it is important to embed them within the long term planning (Cloutier et al., 2018; Simíc et al., 2017) and community structure of the neighbourhood to ensure continuity (Murota, 2014).

## Conclusion and future directions of research

Given the scale of action required to address climate change in cities, neighbourhoods emerge as the next frontier in urban climate action research. Neighbourhoods have the potential to locally respond to the global problems of climate change. Their unique scale, located at the intersection of the city and the individual/building, affords them with multiple opportunities to stir collective climate action. Proximity and tangibility of participating in climate action encourages neighbourhood residents to collectively address mitigation and adaptation challenges. Neighbourhood climate action builds upon the rich literature and practice around neighbourhood renewal (Rohe, 2009; Rowlands, 2011) and sustainability (Grazieschi et al., 2020; Luederitz et al., 2013) by underlining the new skills (Cloutier et al., 2018), tools (Pulselli et al., 2018) and constellations (Aylett, 2013) needed to address climate change at the neighbourhood scale. Neighbourhood climate action further provides a contextual boundary and analytical sharpness to bottom-up action for sustainability (Seyfang & Haxeltine, 2012; Seyfang & Smith, 2007) by bringing forth the unique planning mechanisms (Bradley et al., 2017; Rohe, 2009), motivations (Kwok et al., 2019; Ruef & Kwon, 2016) and challenges (Aylett, 2013; Pulselli et al., 2018) of the neighbourhood scale.

Existing scientific literature provides multiple examples of successful neighbourhood scale projects from neighbourhood greening, infrastructure mapping to energy upgrades. However, challenges remain in terms of social cohesiveness, resources, power and continuity to scale up and magnify neighbourhood climate action. In this paper, we have provided a broad conceptualisation to the concept of neighbourhood climate action. This can serve as a basis for future empirical analysis of neighbourhood climate action efforts as well as form a basis for focused systemic reviews. Here, we present four future venues of research and action to address these challenges and gaps in knowledge:

- A socio-technical-ecological conceptualisation of the neighbourhood: The neighbourhood emerges as a complex entity with physical components like buildings, roads and other built infrastructure, a social network of people and ecological network of trees, plants, drains and other natural components. Quantitative research largely views neighbourhood is a physical entity whereas qualitative research addressed its social component in terms of special interest groups and residents, their networks and relationships. There is a need to develop a mix of technical, social and ecological criteria and scenarios through a mixed research designs where tangible aims like reduced GHG emissions also address intangible benefits like community building and social learning. This is also an opportunity to create neighbourhood scale data support systems that can help design locally relevant climate action projects. Given that neighbourhood governance systems have diverse participating actor, communicating climate data to multiple groups also requires further exploration.
- Socio-economic variables: Multiple papers in this review have indicated that neighbourhood climate action is often weakest in the most vulnerable neighbourhoods (Gilderbloom et al., 2017; Guardaro et al., 2020; Hendricks et al., 2018; Passe et al., 2020). Neighbourhoods that exhibit social and physical vulnerability to climate change often do not have the resources or time to participate in engagement activities around adaptation and resilience building (Meyer et al., 2018). This brings forth the socio-economic variability across neighbourhoods and their capacity to address climate change. Further, our review was limited to the socio-economic context of the Global North. Comparing and contrasting these findings with cities and neighbourhoods located in the Global South will help broaden the conceptualization of neighbourhoods, their governance structures and possibilities of interventions.
- Temporal continuity: The small scale and local nature of neighbourhood scale projects is often short lived. There are opportunities to ensure continuity by exploring ways of translation of city level climate goals into neighbourhood action plans (Cloutier et al., 2018; Hirsch et al., 2011). A longitudinal replication of case studies and projects can provide insights into how action research or experimentation projects sustained climate action. If not, what governance or institutional structures could facilitate this? Further, rather than existing as silos, do projects integrate with the multi-level governance structures?
- Capacity development: Locating climate action at the neighbourhood demands development of new skills and capacities of stakeholders for addressing the issue. These range from learning to work as a group to collecting and understanding locally relevant data. Similarly, an exploration into the devolution of power at the neighbourhood scale, in different political contexts, to ensure that participation goes beyond top-down consultation needs further exploration.

As cities prepare to re-imagine their structure in a post pandemic world as well as be equal partners in the action against climate change, the scale of the neighbourhood promises to be an exciting venue for research and practice.
